# Evaluating stress experienced by caregivers of children with special health care needs via biomarkers: A systematic review

**DOI:** 10.1097/MD.0000000000044177

**Published:** 2025-09-05

**Authors:** Aline Cristiane Cavicchioli Okido, Luis Carlos Lopes-Junior, Wendy Sue Looman, Jaqueline Brosso Zonta, Leticia Patrice Mancini Correa, Mariane Caetano Sulino, Iran Malavazi, Regina Aparecida Garcia Lima

**Affiliations:** a Departament of Nursing. Federal University of São Carlos, São Carlos, São Paulo, Brazil; b University of São Paulo at Ribeirão Preto College of Nursing, Ribeirão Preto, São Paulo, Brazil; c Departament of Nursing, Federal University of Espírito Santo, Vitória, Espírito Santo, Brazil.; d University of Minnesota School of Nursing. Minneapolis, MN

**Keywords:** alpha-amylase, biomarkers, caregivers, child, cortisol, pediatric nursing, stress

## Abstract

**Background:**

The body of literature on physiological measures of stress in caregivers of children with special health care needs (CSHCN) is emerging; however, a nondisease-based review of this literature has not yet been conducted. This study aimed to synthesize and analyze scientific evidence available in the literature on biomarkers associated with stress in caregivers of CSHCN.

**Methods:**

We conducted a systematic review of studies published in 7 electronic bibliographic databases: Embase, MEDLINE/PubMed, Cochrane Library, Web of Science, CINAHL, Scopus, and PsycINFO, with no publication data restrictions. The internal validity and risk of bias of the RCT were assessed using the RoB 2 tool, and for NRCTs, the ROBINS-I was employed. The Newcastle–Ottawa Scale was used to evaluate the internal validity of case–control studies and the JBI tool was used to evaluate cross-sectional studies.

**Results:**

The search identified 755 papers, and 7 articles were selected and included in the analysis. The included studies were conducted across diverse geographic regions: 3 in North America (the United States and Canada), 2 in Europe (the United Kingdom and Croatia), 1 in Brazil, and 1 in South Korea, demonstrating a degree of international representation. Most of the studies (n = 3; 42,8%) were experimental (RCT or NRCT). Regarding critical appraisal, the majority (n = 5; 71.42%) were considered to be of high quality and presented a low risk of bias through specific tools by study design, although 2 were classified as having a high risk of bias. Cortisol was analyzed in all studies, whereas alpha-amylase was measured in only one study, predominantly using saliva samples. Studies have indicated a tendency for lower cortisol levels upon awakening among caregivers of CSHCN children compared to caregivers of healthy children.

**Conclusions:**

This review suggests that there are important differences between caregivers of CSHCN and healthy children regarding biomarker measures. Biomarkers enable objective measurement of stress and may complement self-report measures that are more commonly used in studies of family caregiving. This review underscores the importance of systematically assessing caregivers’ needs in clinical practice and supports the development of public policies and future research initiatives that incorporate biomarker analysis.

## 1. Introduction

Children with Special Health Needs (CSHCN) require greater attention and care from health professionals and their families because of their chronic physical, developmental, behavioral, or emotional conditions.^[1]^ This definition emphasizes care demands rather than medical diagnosis.^[2]^ These care demands include medication administration, enteral diets, glycemic control, psychomotor rehabilitation, and oxygen therapy in addition to routine preventive and developmental care.^[3]^

Continuous care demands extend beyond those routinely required for healthy children, thereby generating stress among caregivers.^[4,5]^ Some of these caregivers provide highly skilled care for up to 24 hours a day, negatively impacting their sleep duration and quality, resulting in excessive fatigue and depressive symptoms,^[6]^ jeopardizing their long-term caregiving capacity.^[7]^

Mothers of children with chronic conditions exhibit significantly higher rates of depression, anxiety, and referrals to secondary mental health services compared to mothers of children without long-term conditions.^[8]^ Therefore, it is imperative to incorporate actions that support caregivers’ mental health needs of caregivers.^[5]^ Additionally, mothers of children with chronic conditions have a significantly elevated occurrence of obesity, hypertension, and back pain compared to mothers of children without long-term conditions.^[8]^

To elucidate the physiological mechanisms associated with the development of diseases among caregivers experiencing stress, study compared perceived stress levels between mothers of healthy children and mothers of children with chronic illnesses, examining 3 biomarkers associated with cellular aging: telomere length, telomerase activity, and the oxidative stress index.^[9]^ According to the findings, within the caregiver group, the longer the duration of caregiving, the shorter the mothers’ telomere length, lower telomerase activity, and higher oxidative stress.^[9]^ Research on telomeres indicates that stress influences physical health through neuroendocrine and inflammatory responses that damage cellular DNA, although repair of such damage is possible through stress healing and recovery.^[10,11]^

According to a scoping review examining the impact of chronic stress associated with caring for a child with a chronic condition, another physiological mechanism linked to stress involves activation of the hypothalamic–pituitary–adrenal (HPA) axis with a particular focus on cortisol, a glucocorticoid hormone responsible for regulating the HPA axis.^[12]^ Generally, an increase in cortisol levels in response to sporadic stress is considered to be a beneficial physiological response. However, elevated cortisol levels over the long-term can lead to a suppressed immune response and increased susceptibility to chronic health conditions.^[12]^

However, despite robust evidence of a psychobiological stress mechanism in health-related outcomes, many stress measures in health literature still rely exclusively on self-report measures. In isolation, these measures are limited because of cultural differences in measurement, reliance on conscious recall bias, variability in experience over time, and calibration effects.^[13]^

Therefore, the incorporation of biomarkers along with self-report measures reflects the importance of adopting a complex system lens to comprehend the dynamic relationships among stress, social contexts, and outcomes in family caregiving.^[14–18]^

Scholars have long endorsed a nonmedical diagnosis-based approach to studying stress among caregivers of children. In 1989, researchers^[19]^ highlight that diagnostic labels explain little about what matters in the lives of CSHCN and their families. More recently, scholars^[20]^ urged exploration of indicators other than child diagnoses to explain caregivers’ health outcomes in these families. Nevertheless, a disease-based approach persists in the emerging research on stress biomarkers in families. Most studies concerning biomarkers in family caregiving have focused on specific conditions, such as caregiving for children with autism^[21,22]^ or for family members with cancer.^[23,24]^

The body of literature on physiological measures of stress in CSHCN caregivers is emerging, but a nondisease-based review of this literature has not yet been conducted. Therefore, to support precision nursing care approaches that incorporate stress biomarkers in family caregiving research and practice, we conducted a systematic review to identify the current state of science in this area. This study aimed to synthesize and critically analyze the literature on biomarkers associated with stress in caregivers of children with special health care needs (CSHCN). The specific objectives were to: identify the biomarkers assessed in this population; describe the data collection protocols used in relevant studies; and compare biomarker levels between caregivers of CSHCN and caregivers of healthy children.

## 2. Methods

### 2.1. Study design

This systematic review reported high compliance with the Preferred Reporting Items for Systematic Reviews and Meta-analyses (PRISMA) Statement.^[25]^ The study protocol was registered in the International Prospective Register of Systematic Reviews (PROSPERO) under the number CRD42021258051 and has been published elsewhere.^[26]^ Based on the PECOS acronym the following research question stands: What scientific evidence is available regarding the levels of stress-related biomarkers among CSHCN caregivers?

### 2.2. Literature search

A systematic search for primary studies was conducted in December 2021 and updated in May 2023 using 7 electronic databases: *MEDLINE – Medical Literature Analysis and Retrieval System Online (via PubMed*), *Cochrane Library; EMBASE; CINAHL – Cumulative Index to Nursing and Allied Health Literature; APA PsycINFO; Web of Science and SCOPUS*. Manual searches were also performed in several sources, including *Clinical Trial; The British Library*, *Google Scholar* and *medRxiv*. Handsearching was carried out in the reference lists and gray literature, seeking additional studies.

The search strategy was carried out by combining controlled descriptors selected in *MESH (Medical Subject Heading*) on MEDLINE/PubMed, *Emtree terms* on EMBASE, *Cinahl headings* on CINAHL as well and APA thesaurus in the PsycINFO database. To expand the search, the controlled descriptors were combined with their respective synonyms AND and OR keywords using the *Boolean operators* AND and OR. Supplementary Material 1 (Supplemental Digital Content, https://links.lww.com/MD/P818) shows the detailed search strategy for 7 databases and other sources.

### 2.3. Eligibility

To be included in the review, studies were required to meet the following criteria: original studies published in full text, English, Portuguese, or Spanish. Publication date restrictions were not applied in order to ensure eligibility. Books, book chapters, theses, dissertations, and abstracts were excluded from analysis. In this systematic review, we included observational and experimental study designs, including cross-sectional, cohort, case–control, ecological, descriptive, and clinical trials.

Studies comprising caregivers of children aged 2 to 12 years were included using the Medical Subject Heading term “Child.” The limited child age range was intended to focus on children during a relatively stable developmental period and to exclude children in infancy and adolescence, which are periods characterized by rapid changes and transitions. Additionally, studies were included if they included any of the following biomarkers of stress: thermal stress markers, such as heat shock proteins; innate immune markers, such as acute phase proteins; oxidative stress markers; and chemical secretions in saliva and urine.^[27]^

The identified studies were exported to the EndNote® reference manager for storage, organization, and exclusion of duplicates. Screening was performed independently by 2 researchers (JBZ and LPCM) based on the titles and abstracts of the studies at the meeting of the pre-established inclusion and exclusion criteria. The Rayyan platform, developed by the Qatar Computing Research Institute, was used as an auxiliary tool for archiving, organizing, and selecting articles (http://rayyan.qcri.org).

The same reviewers independently evaluated the full texts of the retrieved articles to determine whether they met all inclusion criteria. Discrepancies between the reviewers were resolved either by discussion or by a third reviewer (ACCO) in case of lack of agreement, using the Rayyan tool.

### 2.4. Data extraction

Two researchers independently performed data extraction for each study, based on previously published methods.^[28,29]^ The following data were extracted for the synthesis: study characteristics and aims, study population and baseline characteristics, type of exposure, study design, recruitment methods, assessed biomarkers, time of measurement, follow-up, instruments used to measure psychological stress, outcomes, main findings, and conclusions.

### 2.5. Methodological quality assessment

The internal validity and risk of bias of RCT were assessed using the revised Cochrane Risk-Of-Bias tool for randomized trials (RoB 2),^[30]^ which assesses the risk of bias in 5 domains: randomization process, deviations from the intended interventions, missing outcome data, measurement of the outcome, and selection of the reported result. RoB 2 classifies the risk of bias as follows: low risk of bias: low risk of bias for all domains; some concerns: some concerns in at least one domain, but no high risk of bias for any domain; and high risk of bias: high risk of bias in at least one domain or some concerns for multiple domains, substantially reducing confidence in the results.^[30]^ The risk of bias in nonrandomized studies of interventions (ROBINS-I) was used.^[31]^ The ROBINS-I comprises 7 chronologically arranged bias domains (pre-intervention, at intervention, and postintervention), and the domain level and overall risk of bias are classified as low, moderate, serious, or critical.^[31]^

The internal validity and risk of bias of the observational studies were assessed according to study design. The Newcastle–Ottawa Scale was used to evaluate the internal validity of case–control studies.^[32]^ Regarding the NOS scale, a study can be awarded a maximum of 1 star for each numbered item within the Selection and Exposure categories. A maximum of 2 stars was assigned for comparability. The maximum possible score for this scale is 9 points. The more stars the study received, the better the methodological quality and, therefore, the lower the risk of bias in this study.^[32]^ The *JBI* tool for analytical cross-sectional studies was used to assess cross-sectional studies.^[33]^

For all tools (RoB 2, ROBINS-I, NOS and JBI Critical Appraisal Checklist for Prevalence Data), the same 2 reviewers (ACCO and LCLJ) independently assessed the risk of bias for each included study. Discrepancies were resolved through discussion at each stage, and a consensus was reached.

### 2.6. Data analysis

Owing to the heterogeneity of the studies regarding the condition of CSHCN, the biomarker evaluation protocol and follow-up meta-analysis were not feasible; therefore, the data were presented from a qualitative synthesis.

## 3. Results

### 3.1. Study selection

**Figure 1.**
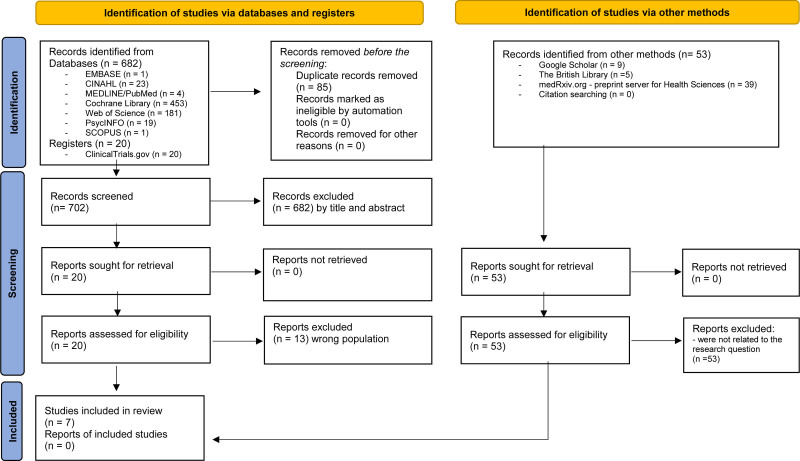
PRISMA flowchart for study selection. PRISMA = preferred reporting items for systematic reviews and meta-analyses.

In Figure 1, the search identified 755 papers (i.e., 682 retrieved studies from databases plus registers and 53 retrieved studies from other sources). Among the studies, 85 were duplicated in electronic databases and were excluded. After analyzing the titles and abstracts, 682 articles were excluded; therefore, 20 articles that met the inclusion criteria were included in the full-text analysis. A complete reading was then performed, but 13 studies were excluded because of incorrect population assessments. The main reason for exclusion in these reports was the inclusion of children aged > 12 years. 7 articles were selected for the data extraction and critical appraisal.

### 3.2. Characteristics of the studies

A total of 528 caregivers of CSHCN participated in the study, of whom 415 (78.59%) were mothers, with a mean age of 38.08 years old. Regarding the year of publication, the articles were concentrated between 2011 and 2020, and no previous studies on this topic were identified.

The included studies were conducted across diverse geographic regions: 3 in North America (the United States and Canada), 2 in Europe (the United Kingdom and Croatia), 1 in Brazil, and 1 in South Korea, demonstrating a degree of international representation.

The field of psychology has led to publications,^[34–37]^ and only one study has been conducted on nurses.^[37]^ Regarding the methodological design used, 2 studies were case–control,^[35,38]^ 3 were RCT,^[34,36,39]^ 1 was a cross-sectional observational study^[40]^ and 1 was an NRCT.^[37]^ Table 1 shows the chronological characteristics of the 7 studies included in this systematic review.

**Table 1:** Characteristics of the included studies.

Citation/country	Aim	Study design	Participants	Biomarkers evaluated	Protocol and follow-up	Instruments assessed	Main findings
Bella et al, 2011/Brazil	Evaluate the level of salivary cortisol and perceived burden, stress, and health of mothers and primary caregivers of children (4–11 yr of age) with cerebral palsy	Case–control	75 mothers of children with cerebral palsy Case group: 37 Control group: 38	Salivary Cortisol	Four samples in 1 d (after waking up, 30 min later, before lunch and before dinner).	Perceived stress questionnaire Burden Interview Short Form Health Survey	The cortisol concentrations were lower in the study group mothers. Both groups showed the diurnal salivary cortisol rhythm preserved. The mothers of children with cerebral palsy with lower income had lower salivary cortisol levels than those of the control group who live in even worse socioeconomic conditions.
Fecteau et al, 2017/Canada	Describe diurnal cortisol activity and perceived stress by the parents before and during the service dog’s presence in the family.	RCT	98 caregivers (85 mothers and 13 fathers) of children with Autism spectrum Disorder Experimental group: 49 families Control group: 49 families	Salivary Cortisol	Three samples 1 d/wk (after waking, 30–45 min later and before bedtime) for each of the 15 wk: 3 wk prior to the dog’s arrival in the family and during the 12 wk following the dog’s introduction.	The Parenting Stress Index-Short form	Fathers had higher awakening cortisol response as well as morning cortisol levels than mothers with ASD. Parents of children with ASD showed low morning cortisol activity similar to hypocortisolism. Significant reductions in awakening and morning cortisol levels during the first 12 wk of the dog’s presence in the family. Parents with the highest morning cortisol prior to the dog’s arrival showed the largest decrease at posttest.
Padden and James, 2017/United Kingdom	Compare physiological markers of stress between parents of children with and without ASD.	Case–control	76 parents (mother–father dyads) of children with Autism spectrum Disorder Control: 38 Case group: 38	Salivary cortisol and alpha-amylase	Four salivary cortisol samples in 1 d (after waking up, 15, 30 and 45 min later). One salivary alpha-amylase at 12:00.	Parenting Stress Index-Short Form Hospital Anxiety and Depression Scale Parental Responsibility Scale Brief COPE Short Form Social Support Questionnaire Pittsburgh Sleep Quality Index	Parents in the ASD group had significantly lower cortisol levels on the third morning measure than parents in the control group. The sAA not differ significantly among the groups. Likewise, no significant differences were observed between mothers and fathers.
Choi and Kim, 2018/South Korea	Investigate the effects of the Thank you–Sorry–Love (TSL1) program on posttraumatic growth (PTG) and cortisol level in parents of children with cancer	RCT	15 mothers of children with cancer Experimental group: 7 Control group: 8	Plasma cortisol	Three blood samples at 10am (1 wk before the TSL Program, 1 and 10 wk after.	Posttraumatic Growth Inventory	The TSL program affected cortisol levels at the posttest, but the effects were not maintained at the follow-up.
Da Paz et al, 2018/United States	Evaluate the effects of WD (written disclosure) on caregiver psychophysiological stress	RCT	71 primary caregivers of children with Autism spectrum Disorder Experimental group: 33 mothers, 3 fathers Control group: 33 mothers, 2 fathers	Salivary Cortisol	Two samples (after waking up and 30 min after) across 2 consecutive days (before the intervention and 6 mo after.	Perceived Stress Scale Caregiver Strain Questionnaire Parenting Stress Index-Short Form	The cortisol awakening response was significantly lower in the treatment group compared to controls at 6-mo follow-up.
Ljubičić et al, 2020/Croatia	Investigate awakening cortisol indicators and to explore their association with stress perception, advanced glycation end products (AGEs), depression and anxiety in parents of children with chronic conditions	Cross-sectional	146 parents of children with several diagnosis (Down syndrome, autism spectrum disorder, cerebral palsy, diabetes mellitus type 1) Mothers: 93 Fathers: 53	Salivary Cortisol	Five samples (before bedtime, after waking up, 15, 30 and 60 min later).	Child’s Challenging Behavior Scale The Parental Stress Scale The Perceived Stress Scale The Generalized Anxiety Disorder Scale The Patient Health Questionnaire The Brief Resilience Scale The Beach Center Family Quality of Life Scale	The circadian rhythm of awakening cortisol was preserved in all study groups. Parents of children with a chronic condition had lower cortisol awakening response compared to the parents of healthy children. Parents of children with DS and DMT1 showed reduced AUC_I_, while ASD parents had a borderline insignificant result in the regression analysis.
Roberts et al, 2020/United States	Investigate the effectiveness of mindfulness-based stress reduction (MBSR) at reducing parent stress, as measured by both psychological self-report and a physiological biomarker.	NRCT	47 parents of children with Developmental delay Mothers: 43 Fathers: 4	Salivary Cortisol	Four samples for each of pretest, posttest and 6-mo follow-up (after waking up, 30, 45 and 60 min later	Parenting daily hassles	There was also a significant reduction in salivary cortisol (AUC) at the 6-mo follow-up.

ASD = autism spectrum disorder, AUC = area under curve, DMT1 = diabetes mellitus type 1, DS = Down Syndrome., NRCT = nonrandomized controlled trial, RCT = randomized controlled trial, sAA = alpha-amylase.

### 3.3. Study population and baseline characteristics in the included studies

Among the diagnoses made by children, autism spectrum disorder (ASD) has been the most studied.^[34–36,40]^ One study included patients with different chronic conditions.^[40]^ With regard to the eligibility criteria, 3 studies excluded participants who used steroid-based medications.^[34,38,40]^ In one study, night work, breastfeeding, and pregnancy were considered as exclusion criteria.^[39]^ Two studies focused exclusively on mothers.^[38,39]^ Five additional studies included fathers; however, mothers accounted for approximately 80% of the total sample across all studies.^[34–37,40]^

Of the 3 case–control studies, only one described the criteria for pairing/matching the experimental and control groups.^[35]^ In this study, the authors recruited a control group of families with a typically developing child of the same sex and aged within 18 months of a matched child with ASD. Parents in the control group were also required to be aged within 10 years of the matched parent in the ASD group.

One study did not report the pairing criteria, although the control and study groups were similar in terms of the ages of the mothers and children.^[38]^ The number of children in the family was considered as a factor in the analysis.^[38]^ Other reported no details regarding matching, and the control group was younger than the study group.^[40]^

### 3.4. Biomarkers assessed and protocol

Most of the biomarkers evaluated in these studies used saliva samples, whereas only one study used plasma samples.^[39]^ Cortisol was analyzed in all studies, whereas only one study analyzed alpha-amylase.^[35]^

The data collection procedures varied among the studies, ranging from collecting samples on a single day^[35,38,40]^ to weekly collections over 15 weeks.^[34]^ All studies collected the first morning sample upon awakening, and the number of subsequent samples varied from 1 to 4 daily. Salivary cortisol concentrations have been reported in different units: μg/dL,^[34–36,38]^ mL/dL^[39]^ and nmol/L.^[40]^

All studies applied at least one subjective scale to measure the psychosocial and emotional aspects of the caregivers. Among the instruments, the most adopted was the Perceived Stress Scale, which was used in 3 studies^[36,38,40]^ and the Parenting Stress Index-Short Form.^[34–36]^

Sample exclusions owing to insufficient quantities have been reported.^[35–37]^ Two studies reported the exclusion of samples that did not comply with the saliva collection guidelines, where samples were excluded if there were more than 10 minutes^[35]^ or 15 minutes^[40]^ differences between the reports of waking and collecting the first sample.

Four studies implemented an intervention involving family members. One was called the TSL Family Program (Thank you–Sorry–Love).^[39]^ This intervention was conducted by a social worker and consisted of 12 weekly sessions lasting approximately 90 minutes each. The objective was to promote the expression of positive emotions such as gratitude, empathy, and guilt relief among family members. In contrast, another intervention included a service dog in the family.^[34]^ The “Written Disclosure” intervention asked family members to write for 20 minutes a day for 3 days about a traumatic situation they experienced.^[36]^ Finally, 1 intervention employed mindfulness exercises for 8 weeks.^[37]^

### 3.5. Main findings and practice implication

The studies exhibited significant variations in sampling procedures, inclusion criteria, cortisol response assessments, and objectives. Overall, the studies identified a tendency for lower levels of cortisol upon awakening among family members of children with CSHCN compared to family members of healthy children. Nevertheless, the diurnal cortisol rhythm remained preserved across all groups, with no statistically significant differences observed between family groups. One study reported a significant difference in stress biomarker levels between mothers and fathers.^[34]^ Specifically, fathers exhibited higher awakening and morning cortisol levels compared to mothers (*P* < .05).

The effect of family income on cortisol concentration has been reported in only one study.^[38]^ In this investigation, mothers of children with cerebral palsy who live in more challenging socioeconomic conditions and bear the responsibility of caring for a disabled child had high levels of stress. This stress reaches a magnitude that impairs the function of the hypothalamus–pituitary–adrenal cortex axis and has negative repercussions on specific aspects of physical and psychological well-being.

The mindfulness exercise-mediated intervention showed favorable long-term results in terms of a significant reduction in salivary cortisol at the 6-month follow-up, indicating less reactivity to stress.^[37]^ Similarly, researchers^[36]^ indicated that the cortisol awakening response was significantly lower in parents who wrote about personal upsetting incidents (treatment group) compared to controls at the monthly follow-up (*P* < .05). In the TSL program, the results showed significantly improved cortisol levels in the experimental group in the posttest, but the effects of the intervention were not maintained in the follow-up test.^[39]^

Four studies demonstrated significant correlations between self-reporting and physiological measures.^[36,37,39,40]^ One noted concordance between objective and subjective stress measures, indicating that mindfulness-based stress reduction was effective in reducing the frequency of daily parenting hassles and morning cortisol levels.^[37]^ Total cortisol level was also significantly associated with anxiety and depression (*P* = .004 and *P* = .034, respectively), whereas its association with general stress was not statistically significant (*P* = .088).^[40]^

In contrast, no significant correlations between self-reporting and physiological measures were identified in 3 studies.^[34,35,38]^ For example, the presence of a dog resulted in a significant reduction in the perception of stress related to the PCDI (*P* < .01); however, there was no change in cortisol over the 12-week period for the entire cohort.^[34]^

Among the limitations presented, the relatively small sample size^[35–37,39,40]^ and samples collected by participants at home without researcher supervision are highlighted.^[38,40]^

Regarding the recommendations for practice presented by the authors, the importance of considering the evaluation of physiological markers together with self-report instruments^[35]^ was emphasized. Furthermore, greater attention should be paid to the physical and mental health of mothers and/or family caregivers when planning care for these children^[35,36,38]^ through systematic interventions that can prevent or alleviate the daily stress of these family members.^[37]^ The incorporation of family focused care based on the family centered care approach model^[34,38]^ is also recommended.

### 3.6. Risk of bias

**Figure 2.**
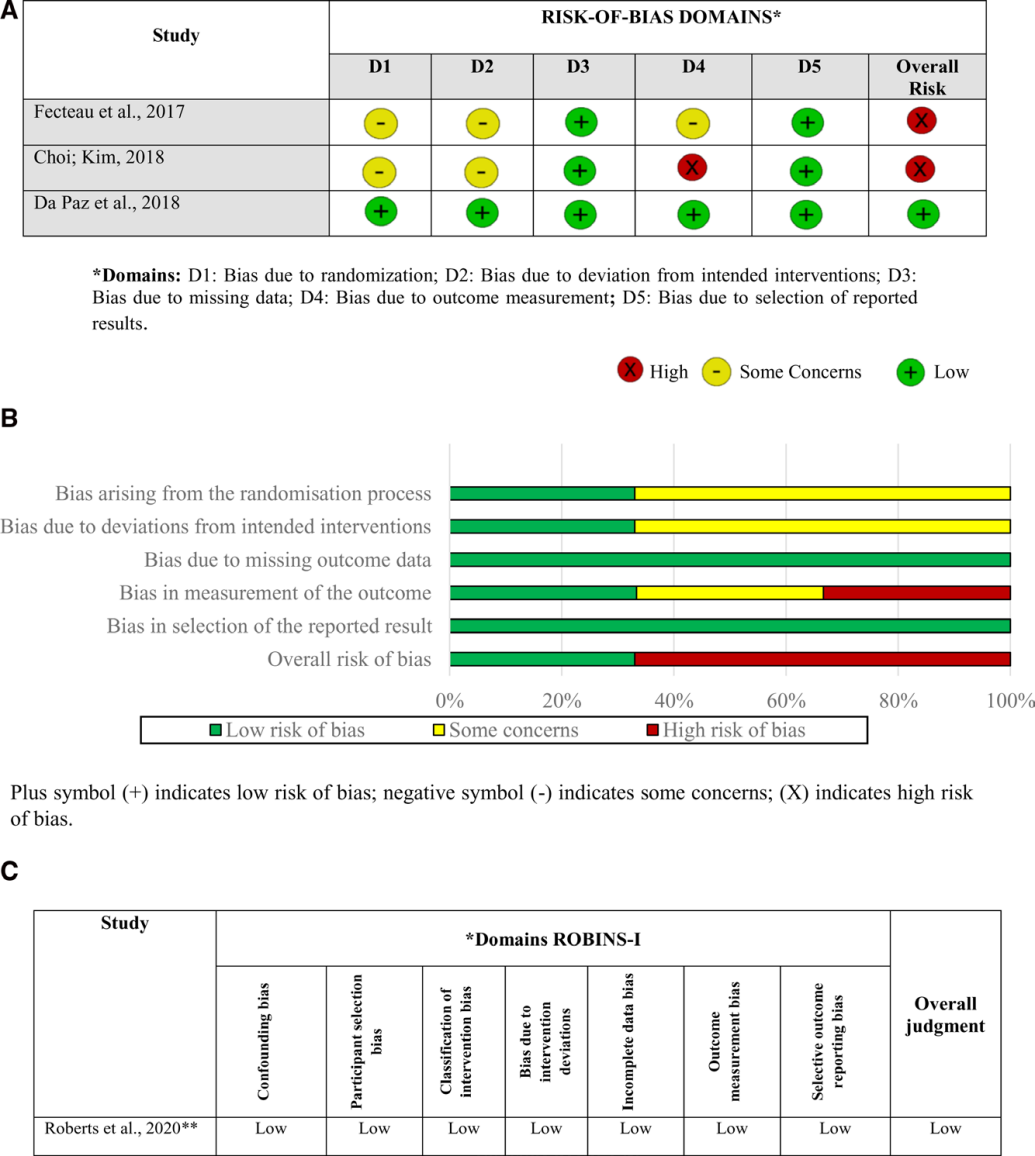
(A) Internal validity and risk of bias assessment of clinical trials according to the RoB 2. (B) Percentage of risk of bias among trials by domains of the RoB 2. (C) Evaluation by consensus of ROBINS-I for nonrandomized controlled trials. ROBINS-I = risk of bias in nonrandomized studies of interventions.

The internal validity and risk of bias of the RCT were assessed using the revised Cochrane RoB 2 (Fig. 2A and B). Among the 3 RCT, 2 (66%) had a high risk of bias^[34,39]^), whereas one study^[36]^ exhibited a low risk of bias, with the 5 domains of RoB 2 showing a low risk of bias. The 2 studies that were classified as having a high risk of bias had some concerns regarding the randomization process and bias due to confounding factors from the intended interventions or bias due to outcome measurement. All 3 studies reported dropouts and therefore had a low risk of attrition bias in this domain of RoB 2. The analysis of the risk of bias in nonrandomized studies of intervention using the ROBINS-I tool (Fig. 2C) indicated that the one study^[37]^ demonstrated a low risk of bias according to the ROBINS-I tool for all 7 domains.

Tables 2 and 3 show the critical appraisal of observational studies. Regarding the methodological quality of case–control studies assessed using the Newcastle–Ottawa Scale,^[32]^ we verified that both studies^[34,38]^ were classified as having high methodological quality, receiving 8 and 9 stars, respectively.

**Table 2:** Methodological appraisal of observational studies. Case–control studies assessed using the Newcastle–Ottawa Scale.

NOS									
Study	Selection				Comparability	Exposure			Score
	Is the case definition adequate?	Representativeness of the cases	Selection of controls	Definition of controls	Comparability of cases and controls on the basis of the design or analysis	Ascertainment of exposure	Same method of ascertainment for cases and controls	Non-response rate	
Bella et al, 2011	★	★	★	★	★	★	★	★	^b^8/9
Padden and James, 2017	★	★	★	★	★★	★	★	★	^b^9/9

b = A study can be awarded a maximum of one star for each numbered item within the selection and exposure categories. A maximum of two stars was assigned for comparability. The maximum possible score for this scale is nine points; NOS = Newcastle-Ottawa Scale.

**Table 3:** Methodological appraisal of observational studies. Cross-sectional studies assessed using the JBI tool.

JBI tool for cross-sectional studies	Were the criteria for inclusion in the sample clearly defined?	Were the study subjects and the setting described in detail?	Was the exposure measured in a valid and reliable way?	Were objective, standard criteria used for measurement of the condition?	Were confounding factors identified?	Were strategies to deal with confounding factors stated?	Were the outcomes measured in a valid and reliable way?	Was appropriate statistical analysis used?
Ljubičić et al., 2020	Y	Y	Y	Y	Y	Y	Y	Y

Y = yes.

## 4. Discussion

This review provides a synthesis of research on stress-associated biomarkers among caregivers of children with special needs and has the potential to assist professionals in directing interventions based on the presented circadian pattern. This review provides opportunities for future interventional research.

In this review, it appears that the focus on biomarkers of stress in caregivers of children with special healthcare needs has emerged in recent decades; however, the number of studies is still insignificant compared to research conducted on caregivers of adults or older people. Scholars^[41]^ conducted an integrative review of salivary biomarkers described in studies related to pain assessment in patients undergoing painful procedures or with painful conditions, and found that 63.6% of the studies were conducted with adults. The analysis of biomarkers such as salivary cortisol has also been applied in studies with healthcare professionals to verify the correlation between occupational activities and stress.^[42]^

Regarding the type of biomarker used for stress assessment, cortisol levels were most often reported in the studies included in this review. Cortisol is the main glucocorticoid frequently used by researchers as a stress biomarker because it reflects the activity of the HPA axis. It has been hypothesized that prolonged exposure to stress (both mental and physical) leads to decreased sensitivity of the HPA axis, resulting in reduced cortisol production by adrenal glands.^[43]^

According to a systematic review of studies evaluating physiological stress measures among parents of children with ASD, salivary cortisol was also the most commonly employed measure.^[22]^ Although salivary cortisol was the most prevalent biomarker in the present systematic review, a variety of data collection procedures as well as different analysis parameters and units of measurement to calculate biomarker concentrations were observed. This finding hinders future comparative analyses between studies, highlighting the importance of standardization.

Salivary alpha-amylase (sAA) was assessed in only one of the included studies. This finding is consistent with the conclusions of a previous review highlighting sAA as a potentially viable biomarker of autonomic nervous system (dys)function in both health and disease.^[44]^ Despite growing interest in sAA within stress research, its use in behavioral medicine studies remains relatively limited. According the authors,^[44]^ the combined assessment of hypothalamic–pituitary–adrenal (HPA) axis and autonomic nervous system activity in stress-related research and interventions may enhance our understanding of the interplay between these systems and their influence on behavioral and health outcomes in clinically relevant populations.

Among the 7 studies included in the review, 2 were exclusively conducted on mothers, whereas the remaining studies included fathers. However, only one study^[35]^ had a similar percentage of fathers and mothers, whereas paternal participation was relatively low in other studies. This result reinforces the view of the female figure as the primary caregiver for children with special health needs, thereby marginalizing the impact of this condition among fathers. However, according to a Spanish study that aimed to explore the perceptions and experiences of parents of children with disabilities, many fathers had to reduce or readjust their work schedules or forego leisure activities and physical exercise, which negatively affected their emotional well-being.^[45]^

ASD prevailed among the analyzed studies. The quest to understand the outcomes of this condition among parents can be explained by an observational study conducted in Italy that aimed to investigate the relationship between caregiver burden levels and the characteristics of different types of developmental disorders.^[46]^ According to the results of this study, caregivers in the ASD group experienced higher levels of burden than parents of children with attention-deficit hyperactivity, language and/or learning, and developmental coordination disorders.

A single study in this review was published in a nursing journal.^[37]^ However, the use of biomarkers supports precision nursing care through standardized approaches for the quantification of stress responses. Incorporating biomarkers into nursing research on families of children with chronic health conditions would contribute to the advancement of science in this area. Including biomarkers, such as salivary cortisol, when standardized across protocols could enable collaboration across research studies, particularly when they are linked with common data elements and standard patient-reported outcome measures.^[47]^ There is a critical need for nursing research and practice to guide the selection and application of stress biomarkers in caregivers of CSHCN patients. Although serial, self-administered saliva collection of cortisol as a biomarker of stress has been shown to be feasible and acceptable in home-based settings,^[48]^ there continues to be low uptake of these tools in nursing research and practice. Slow progress in the field has been marked by limited expertise, skepticism about the usefulness of such parameters, and concerns about cost and participant burden.^[49]^

Nurses are uniquely situated to integrate biomarker measures into research on the effects of family based interventions on outcomes for caregivers and CSHCN. Researchers^[50]^ argued that integrating biomarker measurements into research protocols is a powerful tool for nurses to advance caregiving science across their lifespan. Through precision nursing, they asserted that including biomarkers will enable nurses to more effectively implement intervention research that is hypothesis driven, tailored, and effectively measured. Furthermore, the use of biomarkers in nursing research enables the prediction of the best health outcomes through personalized interventions that link a patient’s omics features with contextual characteristics.^[28,51]^

Regarding the level of evidence of the studies evaluated here, we found that most of the included studies had high methodological quality based on the specific tools per study design. Notably, regarding RCTs, most of them were studies with small samples, different protocols, and short follow-up periods, especially with methodological weaknesses, such as selection and measurement bias. This finding was consistent with that reported by other authors.^[52]^

Hence, our findings indicate that studies with CSHCN caregivers that assess the response of biomarkers associated with stress still require studies with larger samples, better design (i.e., with a low risk of bias), and longer follow-up. In future studies, it is important to consider rigorous analyses of the sensitivity and specificity of the evaluated biomarkers.

This review has the potential to support future intervention-based research focused on family centered care as a parameter for evaluating the effectiveness of proposed actions. According to an observational study conducted in Brazil involving 100 mothers of children with special healthcare needs, care based on the family centered care model was statistically associated with lower physical, social, and emotional burdens on mothers.^[53]^ Furthermore, when the primary focus of attention is not the illness itself but the individual and their family, improvements are observed in access to healthcare services, fewer hours of direct caregiving, and a reduction in financial burden, factors that certainly alleviate stress.^[54]^

Four studies reported significant correlations between self-reported and physiological measures, whereas 3 studies found no concordance between objective and subjective assessments. For instance, a study conducted in India with parents of children with neurodevelopmental disorders – using self-report scales alongside blood testing to evaluate biomarkers – identified a moderate correlation between self-reported stress and biomarker measures (*r* = .481, *P* = .001), suggesting a potential link between caregiver stress, health status, and stress- and immune-related biomarkers.^[55]^ In contrast, a case–control study comparing family caregivers of children with chronic conditions to those of healthy children found no significant correlation between cortisol levels and perceived stress.^[56]^

Although the results of this study support the use of biomarkers by caregivers, it is important to acknowledge some limitations. First, the review was constrained by its focus on studies involving caregivers of children aged 2 to 12 years, potentially overlooking numerous biomarker studies in other age groups. Another limitation is the omission of a search on Biorxiv, an important preprint repository for biological sciences. Additionally, the small sample size (n = 7) limits the strength and generalizability of the conclusions drawn. An important limitation of this review is the absence of a formal assessment of the certainty of evidence using the GRADE (Grading of Recommendations, Assessment, Development and Evaluation) approach. Although our original protocol anticipated the potential use of GRADE, the substantial heterogeneity across the included studies – regarding study design, population characteristics, types of biomarkers assessed, outcome measures, and data collection protocols – precluded the conduct of a meta-analysis. Given that GRADE relies on pooled effect estimates and comparative synthesis, its application was deemed methodologically inappropriate in this context. This decision is in accordance with the Cochrane Handbook for Systematic Reviews of Interventions, which advises against the use of GRADE when statistical synthesis is not feasible or when effect estimates cannot be reliably generated from the available data.^[57]^ Therefore, the findings of this review should be interpreted with caution and primarily considered hypothesis-generating. Nevertheless, these limitations do not negate the findings of the current research but underscore the need for future studies.

## 5. Conclusions

This review of existing literature on biomarkers associated with stress in CSHCN caregivers suggests that there are important differences between caregivers and noncaregivers regarding biomarker measures. Because biomarkers enable objective measurement of stress, they may complement self-report measures that are more commonly used in studies of family caregiving. The paucity of nursing studies in this area presents an opportunity for nurses studying families of children with chronic health conditions to incorporate biomarkers into their work.

The findings of this review may inform interventions targeting healthcare professionals and students by emphasizing the importance of systematically assessing the physical, psychological, and social needs of caregivers of children in clinical practice. Such assessments are essential for establishing supportive conditions for caregiving. At the macro-political level, the development of public policies or social support programs tailored the needs of these caregivers is also warranted. Furthermore, the results of this review may guide future research involving biomarker analysis, as it provides an overview of various data collection protocols currently used in the field.

## Author contributions

**Conceptualization:** Aline Cristiane Cavicchioli Okido, Luis Carlos Lopes-Junior, Jaqueline Brosso Zonta.

**Formal analysis:** Aline Cristiane Cavicchioli Okido, Luis Carlos Lopes-Junior, Wendy Sue Looman, Jaqueline Brosso Zonta.

**Investigation:** Aline Cristiane Cavicchioli Okido, Jaqueline Brosso Zonta, Leticia Patrice Mancini Correa, Mariane Caetano Sulino.

**Methodology:** Aline Cristiane Cavicchioli Okido, Luis Carlos Lopes-Junior, Wendy Sue Looman, Jaqueline Brosso Zonta, Leticia Patrice Mancini Correa.

**Validation:** Luis Carlos Lopes-Junior, Mariane Caetano Sulino, Iran Malavazi, Regina Aparecida Garcia Lima.

**Visualization:** Regina Aparecida Garcia Lima.

**Writing – original draft:** Aline Cristiane Cavicchioli Okido, Wendy Sue Looman, Jaqueline Brosso Zonta, Leticia Patrice Mancini Correa, Mariane Caetano Sulino.

**Writing – review & editing:** Aline Cristiane Cavicchioli Okido, Luis Carlos Lopes-Junior, Wendy Sue Looman, Iran Malavazi, Regina Aparecida Garcia Lima.

## Supplementary Material


