# Supplementary material for: Evaluating stress experienced by caregivers of children with special health care needs via biomarkers: A systematic review
Source: Medicine (Baltimore). 2025 Sep 5;104(36):e44177. doi: 10.1097/MD.0000000000044177 (PMC12419422; doi:10.1097/MD.0000000000044177)
Supplement: Supplementary file 1 [file medi-104-e44177-s001.docx]

**Supplementary Material 1**

| **Databases** | **Item searched** |
| --- | --- |
| MEDLINE/PubMed | **P- POPULATION:**  **#1** ((“Caregivers” [MeSH Terms] OR “Caregiver” [All Fields] OR “Caregiver, Family” [All Fields] OR “Family Caregiver” [All Fields] OR “Informal Caregivers” [All Fields] OR “Mothers” [All Fields] OR “Parents” [All Fields] OR “Caregiver Burden” [MeSH terms] OR “Burden, Caregiver” [All Fields] OR “Caregiver Exhaustion” [All Fields]))  **#2** ((“Disabled Children” [MeSH Terms] OR “Children with Disability” [All Fields] OR “Children, Disabled” [All Fields] OR “Handicapped Children” [All Fields] OR “Children, Handicapped” [All Fields] OR “Child, Disabled” [All Fields] OR “Disabled Child” [All Fields] OR “Medically Fragile Children” [All Fields] OR “Children with Medical Complexity” [All Fields] OR “Technology-dependent Children” [All Fields] OR “Children with Complex Chronic Conditions” [All Fields]))  **#3 #1 AND #2**    **E- EXPOSURE:** not applicable  **C- COMPARISON:** not applicable  **O- OUTCOMES:**  **#4** ((“Biomarkers” [MeSH Terms] OR “Marker, Biological” [All Fields] OR “Biological Marker” [All Fields] OR “Biologic Marker” [All Fields] OR “Biomarker” [All Fields] OR “Immune Markers” [All Fields] OR “Markers, Immune” [All Fields] OR “Serum Markers” [All Fields] OR “Markers, Serum” [All Fields] OR “Clinical Markers” [All Fields] OR “Biochemical Marker” [All Fields] OR “Markers, Biochemical” [All Fields] OR “Marker, Biochemical” [All Fields] OR “Glucocorticoids” [MeSH terms] OR “Glucocorticoid” [All Fields] OR “Glucocorticoid Effect” [All Fields] OR “Effect, Glucocorticoid” [All Fields] OR “Glucorticoid Effects” [All Fields] OR “Receptors, Glucocorticoid” [MeSH terms] OR “Glucocorticoid Receptors” [All Fields] OR “Receptors, Glucocorticoids” [All Fields] OR “Glucocorticoid Receptor” [All Fields] OR “Receptor, Glucocorticoid” [All Fields] OR “Cortisol” [All Fields] OR “Salivary Cortisol” [All Fields] OR “Alpha-Amylases” [MeSH terms] OR “Alpha Amylases” [All Fields] OR “Alpha-Amylase” [All Fields] OR “Salivary Alpha-Amylases” [MeSH terms] OR “Salivary Alpha-Amylase” [All Fields] OR “Salivary alpha Amylase” [All Fields] OR “Alpha-Amylase, Salivary” [All Fields]))  **#5 #3 AND #4** |
| Cochrane Library | **P- POPULATION:**  **#1** (Caregivers) OR (Caregiver) OR (Caregiver, Family) OR (Family Caregiver) OR (Informal Caregivers) OR (Mothers) OR (Parents) OR (Caregiver Burden) OR (Burden, Caregiver) OR (Caregiver Exhaustion)  **#2** (Disabled Children) OR (Children with Disability) OR (Children, Disabled) OR (Handicapped Children) OR (Children, Handicapped) OR (Child, Disabled) OR (Disabled Child) OR (Medically Fragile Children) OR (Children with Medical Complexity) OR (Technology-dependent Children) OR (Children with Complex Chronic Conditions)  **#3 #1 AND #2**  **E- EXPOSURE:** not applicable  **C- COMPARISON:** not applicable  **O- OUTCOMES:**  **#4** (Biomarkers) OR (Marker, Biological) OR (Biological Marker) OR (Biologic Marker) OR (Biomarker) OR (Immune Markers) OR (Markers, Immune) OR (Serum Markers) OR (Markers, Serum) OR (Clinical Markers) OR (Biochemical Marker) OR (Markers, Biochemical) OR (Marker, Biochemical) OR (Glucocorticoids) OR (Glucocorticoid) OR (Glucocorticoid Effect) OR (Effect, Glucocorticoid) OR (Glucorticoid Effects) OR (Receptors, Glucocorticoid) OR (Glucocorticoid Receptors) OR (Receptors, Glucocorticoids) OR (Glucocorticoid Receptor) OR (Receptor, Glucocorticoid) OR (Cortisol) OR (Salivary Cortisol) OR (Alpha-Amylases) OR (Alpha Amylases) OR (Alpha-Amylase) OR (Salivary Alpha-Amylases) OR (Salivary Alpha-Amylase) OR (Salivary alpha Amylase) OR (Alpha-Amylase, Salivary)  **#5 #3 AND #4** |
| Embase | **P- POPULATION:**  **#1** (caregiver OR caregiver burden OR caregiver strain OR caregiver burnout OR family caregiver OR mother OR maternal stress OR mother stress OR parents)  **#2** (handicapped child OR disabled child OR disable children OR children with disability OR children with medical complexity OR children with complex chronic conditions OR technology-dependent children)  **E- EXPOSURE:** not applicable  **C- COMPARISON:** not applicable  **O- OUTCOMES:**  **#3** (biological marker OR biomarkers OR biochemical marker OR glucocorticoid OR glucocorticoid receptor OR hydrocortisone blood level OR salivary cortisol OR amylase OR alpha amylase saliva isoenzyme)  **#4** **#1 AND #2 AND #3** |
| Web of Science | **P- POPULATION:**  **#1** ALL=(Caregivers) OR ALL=(Caregiver) OR ALL=(Caregiver, Family) OR ALL=(Family Caregiver) OR ALL=(Informal Caregivers) OR ALL=(Mothers) OR ALL=(Parents) OR ALL=(Caregiver Burden) OR ALL=(Burden, Caregiver) OR ALL=(Caregiver Exhaustion)  **#2** ALL=(Disabled Children) OR ALL=(Children with Disability) OR ALL=(Children, Disabled) OR ALL=(Handicapped Children) OR ALL=(Children, Handicapped) OR ALL=(Child, Disabled) OR ALL=(Disabled Child) OR ALL=(Medically Fragile Children) OR ALL=(Children with Medical Complexity) OR ALL=(Technology-dependent Children) OR ALL=(Children with Complex Chronic Conditions)  **#3 #1 AND #2**  **E- EXPOSURE:** not applicable  **C- COMPARISON:** not applicable  **O- OUTCOMES:**  **#4** ALL=(Biomarkers) OR ALL=(Marker, Biological) OR ALL=(Biologic Marker) OR ALL=(Biomarker) OR ALL=(Immune Markers) OR ALL=(Serum Markers) OR ALL=(Clinical Markers) OR ALL=(Markers, Biochemical) OR ALL=(Glucocorticoids) OR ALL=(Glucocorticoid) OR ALL=(Glucocorticoid Effect) OR ALL=(Glucocorticoid Receptors) OR ALL=(Cortisol) OR ALL=(Salivary Cortisol) OR ALL=(Alpha-Amylase) OR ALL=(Salivary Alpha-Amylase) OR ALL=(Salivary Alpha Amylase) OR ALL=(Alpha-Amylase, Salivary)  **#5 #3 AND #4** |
| Scopus | **P- POPULATION:**  **#1** TITLE-ABS-KEY((Caregivers OR Caregiver OR Caregiver, Family OR Family Caregiver OR Mothers OR Parents OR Caregiver Burden)**)**  **#2** TITLE-ABS-KEY((Disabled Children OR Children with Disability OR Children, Disabled OR Disabled Child OR Medically Fragile Children OR Children with Medical Complexity OR Technology-dependent Children OR Children with Complex Chronic Conditions))  **#3 #1 AND #2**  **E- EXPOSURE:** not applicable  **C- COMPARISON:** not applicable  **O- OUTCOMES:**  **#4** TITLE-ABS-KEY((Biomarkers OR Glucocorticoids OR Cortisol OR Salivary Cortisol OR Alpha-Amylase OR Salivary Alpha-Amylase OR Salivary Alpha Amylase OR Alpha-Amylase, Salivary))  **#5 #3 AND #4** |
| CINAHL | **P- POPULATION:**  **#1** (Caregivers [Cinahl headings] OR Family Caregiver Status [Cinahl headings] OR Caregiver Burden [Cinahl headings] OR Parents of Disabled Children [Cinahl headings] OR Mothers [Cinahl headings])  **#2** (Child, Disabled [Cinahl headings] OR Child, Medically Fragile [Cinahl headings] OR Handicapped Children [Cinahl headings])  **#3 #1 AND #2**  **E- EXPOSURE:** not applicable  **C- COMPARISON:** not applicable  **O- OUTCOMES:**  **#4** (Biologic Markers [Cinahl headings] OR Hydrocortisone [Cinahl headings] OR Alpha-Amylase [Cinahl headings])  **#5 #3 AND #4** |
| PsycINFO | **P- POPULATION:**  **#1** (Caregivers OR Family Caregivers OR Mothers OR Parents OR Caregiver Burden)    **#2** (Disabled Children OR Children, Disabled OR Handicapped Children OR Medically Fragile Children OR Children with Medical Complexity OR Technology-dependent Children OR Children with Complex Chronic Conditions)  **#3 #1 AND #2**  **E- EXPOSURE:** not applicable  **C- COMPARISON:** not applicable  **O- OUTCOMES:**  **#4 (**Biological Markers OR Biochemical Markers OR Biomarkers OR Clinical Markers OR Glicocorticoids OR Cortisol Awakening Response OR Hydrocortisone OR Cortisol)  **#5 #3 AND #4** |
| **Registers** | **Item searched** |
| ClinicalTrial.gov | (Caregivers OR Family Caregiver Status OR Caregiver Burden OR Mothers OR Parents of disabled children OR Caregiver burnout AND (Biomarkers) |
| WHO International Clinical Trials Registry Platform | (Pediatric Oncology Nurse OR Pediatric Oncology Nursing) AND (Professional Competence OR Competency-Based Education) |
| **Organizations and Websites and grey literature** | **Item searched** |
| The British Library (UK) | **P- POPULATION:**  **#1** (Caregivers OR Family Caregiver Status OR Caregiver Burden OR Parents of Disabled Children OR Mothers)  **#2** (Child, Disabled OR Child, Medically Fragile OR Handicapped Children)  **#3 #1 AND #2**  **E- EXPOSURE:** not applicable  **C- COMPARISON:** not applicable  **O- OUTCOMES:**  **#4** (Biologic Markers OR Hydrocortisone OR Alpha-Amylase)  **#5 #3 AND #4** |
| Google Scholar | (“Caregivers” OR “Family Caregiver Status” OR “Caregiver Burden” OR “Mothers”) AND (“Child, Disabled”) AND (“Biomarkers”) |
| Preprints for Health Sciences [medRXiv] | (“Caregivers” OR “Family Caregiver Status” OR “Caregiver Burden” OR “Mothers”) AND (“Child, Disabled”) AND (“Biomarkers”) |
